# SpaSE-UNet3D: Sensor-Driven Wildfire Detection and Progression Prediction from VIIRS Multispectral Imagery

**DOI:** 10.3390/s26165116

**Published:** 2026-08-12

**Authors:** Nikolaos Mavros, Dimitrios Katsaros

**Affiliations:** Department of Electrical and Computer Engineering, University of Thessaly, 38334 Volos, Greece; nmavros@uth.gr

**Keywords:** wildfire detection, VIIRS, active fire, fire progression, 3D U-Net, squeeze-and-excitation, remote sensing, deep learning, satellite imagery, label quality audit

## Abstract

Timely wildfire monitoring depends critically on optical and thermal infrared sensor observations from spaceborne instruments. The TS-SatFire benchmark (2025) consolidates multispectral VIIRS image stacks from Suomi-NPP and NOAA-20 for three tasks: active fire (AF) detection, burned area (BA) mapping, and fire progression (FP) prediction. We make two contributions. First, a systematic label-quality audit reveals that many fires lack ground-truth annotations; 18 training fires and 2 test fires were excluded for AF, and the two unannotated test fires cannot be scored by any model. We further document the benchmark’s scoring procedure, which differs from ours in ways that make the two sets of figures incomparable, and the BA label encoding in the released GeoTIFFs; the BA task is only audited. Second, we propose SpaSE-UNet3D, a spatial squeeze-and-excitation 3D U-Net whose spatial-only (1,3,3) convolutions avoid temporal mixing on short observation windows, while SE channel attention reweights the VIIRS spectral bands dynamically. With micro-averaging over all test pixels, it reaches F1 = 0.8549±0.0005 on AF and 0.3845±0.0221 on FP at TS = 2, matching or exceeding the strongest published baselines on their respective terms. A single-day AF input reaches 0.8520±0.0008, within 0.003 of the two-day figure, indicating that one acquisition carries most of the detectable signal, whereas published baselines use up to six days; on FP, we use one third of their temporal context. An ablation shows the spatial-only design matches the accuracy of a full (3,3,3) network with 2.72× fewer parameters. Code and results are publicly available.

## 1. Introduction

Wildfires are increasingly destructive across temperate and boreal biomes, and timely automated detection of active fire fronts, burned extents, and near-term fire progression is essential for operational response. Satellite-borne optical and thermal infrared sensors are the primary mechanisms enabling this monitoring at continental scale, with daily or twice-daily revisit and spectral bands specifically calibrated for fire radiometry. Among spaceborne systems, the Visible Infrared Imaging Radiometer Suite (VIIRS) aboard Suomi-NPP and NOAA-20 stands out: it provides 22 bands spanning the visible, near-infrared, and thermal infrared spectrum, of which five imaging-resolution bands at 375 m are directly exploited for active fire detection [1]. Barmpoutis et al. [2] survey the full landscape of optical sensor systems for early fire detection, distinguishing terrestrial, airborne, and spaceborne architectures and confirming that VIIRS and MODIS spaceborne sensors provide the most operationally scalable coverage for large-scale wildfire monitoring. Alongside these learned approaches, index-based methods applied to moderate-resolution optical sensors remain widely used for post-fire burned-area assessment: spectral indices such as the normalised burn ratio have been applied to Landsat 8 imagery to delineate burning areas in protected forest [3], and comparable index-based procedures using Sentinel-2 data have been used to quantify burned extents in wildlife sanctuaries over multi-year periods [4]. These methods provide the operational baseline against which learned approaches are measured.

The recently released TS-SatFire dataset [5] consolidates several years of VIIRS observations into a common benchmark for three wildfire tasks: active fire (AF) pixel detection, burned area (BA) mapping, and fire progression (FP) prediction. Each fire event is represented as a temporal stack of multispectral VIIRS sensor images, with up to eight daytime reflectance and thermal channels plus complementary nighttime thermal bands—a design motivated by the demonstrated value of combining daytime optical sensor observations with nighttime thermal infrared data for round-the-clock fire tracking [6]. The dataset paper reports results for 12 baselines ranging from 2D U-Nets [7] to 3D transformer variants [8,9], using sensor temporal stacks of up to TS = 6 days. The strongest published AF baseline is SwinUNETR-3D at TS = 2 with F1 = 0.823; the strongest published FP baseline is U-Net-3D at TS = 6 with F1 = 0.375.

The benchmark has been used to rank twelve architectures against one another, but neither the completeness of its labels nor the evaluation procedure used to produce those rankings has been examined. Both, we find, materially affect the reported ordering. We therefore make two contributions. The first is SpaSE-UNet3D, a spatial-only 3D U-Net with squeeze-and-excitation channel attention, designed for the short observation windows this sensor regime provides: it matches the accuracy of a full 3D convolutional network with 2.72× fewer parameters and trains both tasks in under 15 h on a single GPU. The second contribution arose from evaluating the first. Establishing what the architecture actually achieves required examining how the benchmark scores models and whether its labels are complete, and both turned out to be consequential in their own right: a substantial number of fires carry no annotation, and the scoring convention moves reported figures by more than the margin separating published methods. We report the audit, the protocol and the exclusion lists as contributions in their own right, since fair comparison on this benchmark is not otherwise possible.

We pursue two goals. The first is to characterise the benchmark itself. A systematic audit of every fire in every split reveals many fires with entirely missing labels across all three tasks, including two of the seventeen active-fire test fires; fires without annotation cannot be scored, so any full-split test aggregate is fixed for those fires by the empty-ground-truth convention, not by model behaviour. We document the exclusion lists, the undocumented burned-area label encoding, and the effect of filtering at fire and day level, measuring its consequence for accuracy instead of asserting it.

The second goal is architectural. TS-SatFire’s sensor input is very short: operational and dataset conventions favour windows of 1–2 days for AF and 2–6 days for FP. On such windows, the temporal prior that motivates full 3D convolutions in video [10,11] or volumetric medical imaging [12] is not obviously justified, since there is little inter-day signal to decorrelate. We therefore propose SpaSE-UNet3D, a spatial Squeeze-and-Excitation 3D U-Net that uses (1,3,3) convolutions in every residual block, so that the temporal dimension is preserved and is mixed by no convolution in the network. SE [13] channel attention reweights the VIIRS bands per spatial context, and task-specific variants add an ASPP [14,15] bottleneck and a 27-channel auxiliary input for fire progression.

Under the protocol of Section 6, SpaSE-UNet3D reaches F1 = 0.8549±0.0005 on the 15 annotated AF test fires and 0.3845±0.0221 on all 24 FP test fires, both at TS = 2, using two-day windows where the published baselines use six and training in under 15 h on a single NVIDIA T4 GPU (NVIDIA Corporation, Santa Clara, CA, USA). Because the two sets of figures are computed under different scoring conventions, we report ours on their own terms; on those terms they match or exceed the strongest published baselines while using a two-day window against the baselines’ six (Section 6). The BA task is used for auditing only; we train no BA model.

In summary, we (i) audit label completeness across all tasks and splits and document the burned-area encoding; (ii) determine whether published and independently computed figures measure the same quantity and isolate the effect of each difference; (iii) develop an architecture suited to the short windows the benchmark provides and characterise its cost as well as its accuracy; and (iv) establish the run-to-run variability of both tasks, so that reported differences can be read against measurement noise.

The rest of the paper is organised as follows. Section 2 reviews related work. Section 3 describes the TS-SatFire dataset. Section 4 presents our label-quality audit. Section 5 details the SpaSE-UNet3D architecture and its task-specific variants. Section 6 reports the experimental protocol and results. Section 7 discusses the findings and Section 8 concludes.

## 2. Related Work

### 2.1. Wildfire Remote Sensing with Deep Learning

Early work on active fire detection from satellite imagery used per-pixel thresholds on thermal bands, typically VIIRS band I4 or the MODIS analogue [1,16]. Deep learning approaches in the last five years have moved to segmentation networks operating on multi-band VIIRS or Sentinel-2 stacks [17], and more recently to 3D and transformer architectures that incorporate temporal context [18]. Huot et al. [19] released the Next Day Wildfire Spread benchmark, with a related but coarser problem formulation than TS-SatFire. Across this literature, comparisons are made between architectures on a fixed pipeline, while the pipeline itself—label construction, cropping, and the aggregation of per-pixel counts into a reported score—is described briefly and varies between publications, so nominally identical metrics are not always comparable. We quantify that ambiguity on one benchmark in Section 6.2. Zhao and Ban [5] provide the most comprehensive comparison to date, benchmarking 12 architectures spanning U-Net [7], Attention U-Net [20], UNETR [9], SwinUNETR [8], and recurrent encoders on a unified TS-SatFire benchmark. Their reported figures are the reference point for this paper, and Section 6.2 examines the evaluation procedure that produced them.

### 2.2. Factorised Spatiotemporal Convolutions

Tran et al. [10] and Qiu et al. [11] showed that factorising 3D convolutions into separate spatial (1,k,k) and temporal (k,1,1) stages outperforms full (k,k,k) kernels in video classification, with fewer parameters. SpaSE-UNet3D extends this principle to its extreme on short (T≤2) windows by retaining only the spatial component. Our ablation (Section 6.5) supports the parameter-efficiency half of this claim; it does not resolve an accuracy difference.

### 2.3. Relation to Existing 3D U-Net Variants

SpaSE-UNet3D shares its encoder–decoder skeleton with U-Net-3D [12], so it is worth stating where it departs from the 3D baselines it is evaluated alongside. U-Net-3D and Attention U-Net-3D apply isotropic (3,3,3) convolutions throughout, and UNETR [9] and SwinUNETR [8] attend across the temporal axis at every stage. Both families were designed for volumetric medical data, where that axis is a spatial depth of many strongly correlated slices; on a stack of one or two daily acquisitions it is short and weakly correlated. We therefore remove learned mixing along that axis entirely, combining across days only through global pooling in the SE modules and, for progression, a mean before the output head.

### 2.4. Attention in U-Nets

Squeeze-and-Excitation [13] adds a lightweight channel-wise attention module that globally pools feature maps and produces per-channel gating weights. Roy et al. [21] extended this with concurrent spatial-channel SE for 2D semantic segmentation. Atrous Spatial Pyramid Pooling [14], later refined in DeepLabv3+ [15], aggregates multi-scale context via parallel dilated convolutions in the bottleneck. Both modules are standard in semantic segmentation, but their combination with spatial-only 3D convolutions for temporal-satellite tasks is, to our knowledge, new.

### 2.5. Data Quality in Remote-Sensing Benchmarks

Prior work has documented label noise in land-cover benchmarks [22] and machine-learning test sets more broadly [23], showing that erroneous test labels can reorder benchmark rankings even when the underlying models are unchanged. Moreno-Ruiz et al. [24] document omission and commission errors in the MODIS burned-area products from which TS-SatFire’s burned-area labels are partly derived, so label error in this benchmark is inherited as well as introduced. Wildfire benchmarks are also exposed to a form of incompleteness that is easy to miss: their ground truth is the output of another automated product rather than human annotation, so a fire that the source product failed to detect appears as a scene with no positive pixels, indistinguishable at the file level from a scene where nothing burned, and it trains without error. Our audit is in this tradition.

### 2.6. Sensor Systems for Wildfire Monitoring

Barmpoutis et al. [2] provide a comprehensive survey of optical remote sensing systems—from ground-based infrared cameras to spaceborne VIIRS and MODIS sensors—for early fire detection, establishing the sensor taxonomy within which TS-SatFire sits. Chiang and Ulloa [6] demonstrate that combining the Sentinel-3 SLSTR optical/thermal instruments with the VIIRS day-night band (VIIRS-DNB) enables round-the-clock fire tracking, motivating TS-SatFire’s inclusion of both daytime and nighttime VIIRS channels as model inputs. For burned area assessment, Moreno-Ruiz et al. [24] audit the omission and commission errors of MODIS-derived BA products, confirming that these remotely sensed labels carry systematic spatial and temporal biases that propagate to downstream benchmarks—a finding our own audit of TS-SatFire BA labels corroborates. On the deep learning side, Ghali et al. [25] benchmark CNNs and vision transformers for pixel-level wildfire segmentation from satellite imagery, and Casallas et al. [26] apply a 3D-CNN directly to satellite stacks for wildfire early alert, providing the closest architectural precedent to our SpaSE-UNet3D.

## 3. The TS-SatFire Dataset

TS-SatFire [5] consists of a set of fire events, each represented by a multi-day temporal stack of VIIRS observations. Per event, every day has a VIIRS_Day GeoTIFF with eight spatial bands (six daytime reflectance and thermal bands plus two nighttime thermal bands), and a FirePred GeoTIFF with 19 auxiliary bands. The released rasters carry no band names, but the channel ordering is documented in the dataset publication [5], where the eight VIIRS bands and the nineteen auxiliary bands are enumerated as a single 27-channel sequence. We reproduce the correspondence in Table 1 because it is required to interpret any per-band analysis and is easy to lose when the two rasters are read separately. The auxiliary bands fall into four groups: vegetation indices, current weather, terrain, and forecast weather.

In a probe of 62 fires, only the two precipitation bands (3 and 15) are ever uniformly zero—in 12 and 5 fires respectively—which is expected for events burning through periods without rainfall, and indicates valid measurements, not missing data.

### 3.1. Labels

The VIIRS_Day GeoTIFF contains the label channels for all three tasks, encoded in dedicated bands of the same raster.

#### 3.1.1. Active Fire Labels

Band 7 contains active-fire labels, encoded as VIIRS fire confidence levels [1]. Pixels with finite values ≥7 are active-fire pixels, which we threshold to a binary mask.

#### 3.1.2. Burned Area Labels

Band 8 contains burned-area labels. Zhao et al. [5] describe how the BA labels are constructed—as the union of accumulated VIIRS active-fire detections and NIFC perimeters, with case-by-case manual quality control on the test set. The dataset paper does not document the precise storage convention used in the released GeoTIFFs, which we found necessary to verify before any modelling: in practice, band 8 encodes the burned mask implicitly, with finite values in the range [2119,908,660] corresponding to Julian-day codes or perimeter identifiers and NaN values corresponding to unburned pixels. The correct binary extraction is therefore

(1) $$\mathrm{BA}\left(p\right)=1\left[\;\neg \mathrm{NaN}(\mathrm{band}8(p))\;\right]$$

and not a value threshold, where 1[·] is the indicator function and *p* ranges over the pixels of the raster.

Equation (1) is worth stating explicitly because the natural first attempt fails silently. Band 8 is a floating-point raster whose finite entries are large integers—dates or identifiers—and whose unburned pixels are NaN rather than zero. A reader who assumes the band holds a probability or a class score will apply a threshold such as band8(p)>0.5; because every finite value exceeds any threshold in [0,1] and NaN comparisons evaluate to false, this happens to return the correct mask, and the error goes unnoticed until a different threshold or a different comparison is used. Conversely, converting NaN to zero before comparison—as numpy.nan_to_num does, and as the reference loader does elsewhere—turns unburned pixels into valid zeros and silently changes the class balance. The information carried by band 8 is therefore *presence*, not *magnitude*: a pixel is burned if and only if its entry is finite, and the numeric value encodes when or by which perimeter it burned, not how strongly. Every finite value we observed corresponds to a burned pixel; there are no finite non-burn entries. Any future burned-area or fire-progression work on TS-SatFire must use this encoding, and the derived progression target of Equation (2) inherits it.

#### 3.1.3. Fire Progression Labels

Fire progression labels are not stored directly. They are derived from consecutive BA masks: the FP label for day *T* is the set of pixels burned on day T+1 but not on day *T*,

(2) FP(T)=BA(T+1)∖BA(T).

A window is usable only when both the last input day and the following day have valid (non-NaN) band 8.

### 3.2. Splits

The dataset paper defines the following splits.

#### 3.2.1. AF/BA Splits

The AF/BA split contains 138 training fires, 13 validation fires, and 17 test fires (named historical events, e.g., *Dixie*, *Creek*, *Carr*). Two of the 17 test fires, *Calf Canyon* and *Mosquito*, are 2022 events.

#### 3.2.2. FP Splits

The FP validation set extends the AF/BA list from 13 to 15 fires, adding 22713339 and 23301962; since 22713339 is present in the dataset, FP training has 137 fires rather than 138. 23301962 is absent from the Kaggle distribution, so the replicable FP validation set contains 14 fires. The test set is a separate collection of 24 US_2021 fires identified by geographic codes such as US_2021_CA3451712013120211011.

### 3.3. Spatial Coverage of the Centre Crop

TS-SatFire scenes are supplied at the full extent of each fire event, which in our copy of the dataset is 596×595 pixels for all but one event. As is standard practice in satellite segmentation pipelines, and as the reference implementation also does, we operate on a fixed 256×256 crop rather than the full scene, for memory reasons. A crop of this size retains approximately 19% of the imaged area. The reference implementation places the crop at a 30% offset from the scene origin; we centre it (Section 6.2).

This choice is not neutral, because fires are not centred within their scenes. We quantify the loss by counting, for every labelled positive pixel in each fire, whether it falls inside or outside the crop actually used. The results differ markedly between tasks. For active fire, a mean of 26.8% of labelled fire pixels fall outside the crop. For burned area and fire progression, whose labels cover the accumulated extent of the event rather than the instantaneous front, the mean rises to 56.3%, and for several fires the crop retains no labelled positive pixels at all. The most severe active-fire case loses 86.2% of its labelled pixels; among the progression test fires, four lose more than 87%, and one loses all of them.

The consequence is that for a substantial fraction of events the model is asked to predict a target that is largely absent from its input, and is scored on the discrepancy. This is a property of the evaluation setup, not of any model, and it applies equally to every method evaluated under the same convention. Section 6.4 shows that the fraction of positive pixels lost to the crop predicts per-fire progression accuracy, and is a sufficient explanation for the low-scoring cases without appeal to fire behaviour. We report all results under the cropped protocol for consistency with the published baselines, and identify full-scene evaluation as necessary future work.

## 4. Label-Quality Audit

We audit every fire in every split of every task for label completeness. Missing labels occur at two distinct granularities, and conflating them discards usable data: some fires carry no annotation on any day of their temporal extent, while others are annotated on a subset of days only. We therefore audit and filter at both levels. For each fire, we read the VIIRS_Day GeoTIFF for every available day and test whether the relevant label band contains any finite pixel. A fire with zero finite label pixels across its entire temporal extent carries no supervision and is excluded; a fire annotated on some days is retained, and individual unlabelled windows are rejected instead.

### 4.1. AF Audit

Table 2 summarises the audit for all three tasks.

*Fire level.* Of the 138 fires in the AF training pool, 18 carry no active-fire supervision at all and are excluded: 13 have no VIIRS_Day directory in the released data, and 5 have band 7 entirely NaN across every available day. Two validation fires and two test fires fail the same test: 22712904 has band 7 entirely NaN, and one further validation fire has no VIIRS_Day directory. The two test fires are *Calf Canyon* and *Mosquito*, both 2022 events for which active-fire ground truth was never included in the released data. They cannot be scored by any model, and any aggregate reported over the full 17-fire test set therefore depends for those fires on the convention adopted for empty ground truth rather than on model behaviour (Section 6.2). The audited splits contain 120 training, 12 validation and 15 test fires.

*Day level.* Excluding a fire outright because part of its temporal extent is unlabelled discards usable supervision. We instead retain partially annotated fires and reject individual windows: a window is admitted only if band 7 on its final day—the day to which the AF label refers—contains at least one finite pixel. Applied to the audited fire lists, this rejects 143 training windows, leaving 1719 training windows at TS = 2, each supervised by a verified label.

Without the fire-level exclusion, windows drawn from unannotated fires enter training with an all-negative target, since a NaN band read through the reference loader’s nan_to_num conversion becomes a valid all-zero mask. The model is then supervised to suppress activations on scenes that do contain fire. Section 6.3 measures the size of this effect under our pipeline.

*Sensitivity to the criterion.* The fire-level criterion above is the weakest of the available criteria: a fire is removed only when it carries no supervision at all. A stricter alternative removes any fire whose labelled days fall below a fixed fraction of its temporal extent; at a 50% cut-off, three further fires are removed, leaving 117. We adopt the weaker criterion because day-level filtering already guarantees that every training window is verified, making fire-level removal of partially labelled fires unnecessary and wasteful of supervision.

### 4.2. BA Audit

Table 2 includes the BA audit. Burned-area labels are missing far more often than active-fire labels. Of the 137 fires in the BA training pool, 13 have no VIIRS_Day directory and 18 have band 8 entirely NaN on every day, so 31 carry no burned-area supervision at all; a further 17 are labelled on fewer than half their days. Six of the 14 validation fires and seven of the 17 named test fires likewise carry no burned-area label. Because the two tasks read different bands of the same rasters, the AF and BA exclusion lists overlap but do not coincide: a fire may carry active-fire detections while its burned-area band is empty, and the reverse also occurs. We release both lists in full, since the relevant list depends on the task being trained and a summary of the overlap would not suffice.

In addition to the missing-label audit, we also document the practical encoding of band 8 in the released GeoTIFFs. Zhao et al. [5] describe how the labels are produced (union of accumulated VIIRS active-fire detections and NIFC perimeters, with manual quality control on the test set) but do not specify the storage convention in the released rasters—a small but important detail for downstream users. Empirically, we find that finite values in band 8 span the range [2119,908,660], consistent with Julian-day codes or perimeter identifiers from the MCD64A1 MODIS burned-area product rather than a binary or thresholded indicator. 100% of finite pixels are burned pixels; there are no finite non-burn values. The correct extraction of a binary BA mask is therefore presence-of-finite, as stated in Section 3. Documenting this encoding is a prerequisite for any future BA modelling on TS-SatFire.

### 4.3. FP Audit

Table 2 includes the FP audit. The FP audit is more subtle because FP labels are derived at the window level from consecutive BA masks. A fire is excluded if no usable window can be constructed, i.e., if it has zero consecutive (day *T*, day T+1) pairs in which both days have valid BA. Moreover, 48 training fires and 6 validation fires fail this test. No fire is excluded from the test set: all 24 official test fires are reported throughout. Six of them carry no usable window and therefore contribute no counts under micro-averaging, and the remaining 18 are scored normally. We prefer this to a burn-fraction cut-off on the test population, since any such threshold selects fires on a property of their ground truth and its effect on the aggregate is then difficult to separate from model behaviour.

All exclusion lists are reported in full in the supplementary audit reports released with the repository.

Two conventions require comment, and the first accounts for an apparent discrepancy between this table and the abstract. The AF and BA training pools are listed here as 137 rather than the 138 of Section 3.2, and the validation pools as 14 rather than 13, because fire 22713339 appears in the AF/BA training split but is reassigned to fire-progression validation by the dataset paper, and the audit follows the reassigned splits throughout. Our training pipeline instead retains it in the AF training pool and finds it unlabelled, so it counts 138 fires and 18 exclusions where this table counts 137 and 17. Both accountings leave the same 120 usable training fires, which is the quantity that matters for the experiments; the abstract quotes the pipeline’s figure of 18. Fire-progression exclusions are counted at the window level rather than the band level: a fire is unusable when no consecutive (T,T+1) pair has valid burned-area labels on both days, so the three band-level columns do not apply. All exclusion lists are released in full with the code.

## 5. Method: SpaSE-UNet3D

**Figure 1:**
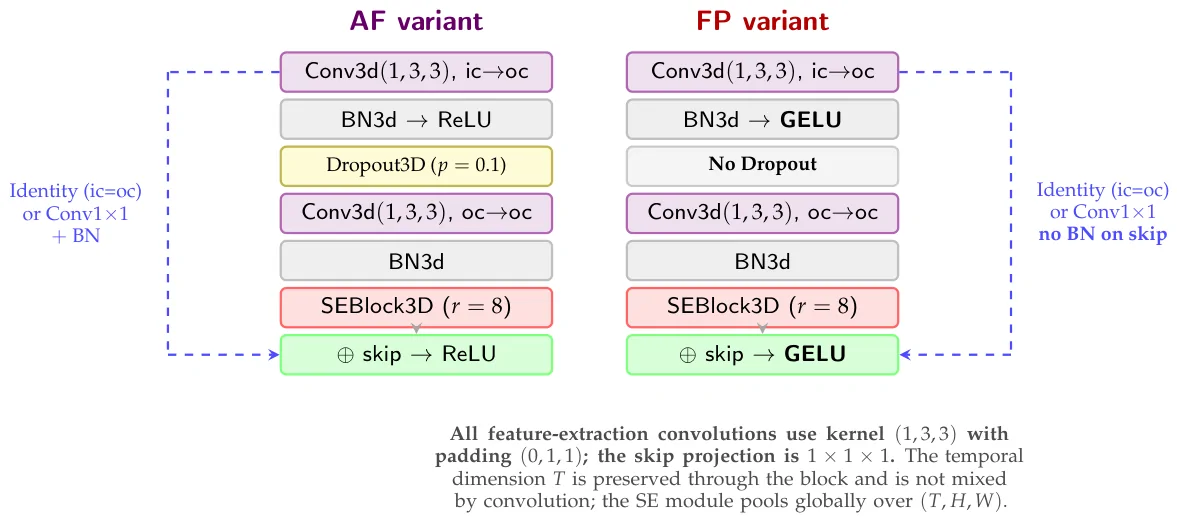
Internal structure of the ResBlock3D used in both SpaSE-UNet3D variants. Both blocks share spatial-only (1,3,3) convolutions and SE channel attention. The AF variant (**left**) uses ReLU activations and Dropout3D for regularisation; the FP variant (**right**) uses GELU and removes Dropout, which empirically hurt convergence on the smaller, sparser FP training set. The skip path either passes the input unchanged (when ic=oc) or applies a 1×1×1 projection convolution. Grey arrows show the forward data path and blue dashed lines the residual (skip) path; the symbol before the word skip is ⊕, element-wise addition. Box fill marks the operation type only: violet, convolution; grey, normalisation and activation; yellow, dropout; red, SE attention; green, residual addition. Bold marks the operations in which the two variants differ; ic and oc are the input and output channel counts.

**Figure 2:**
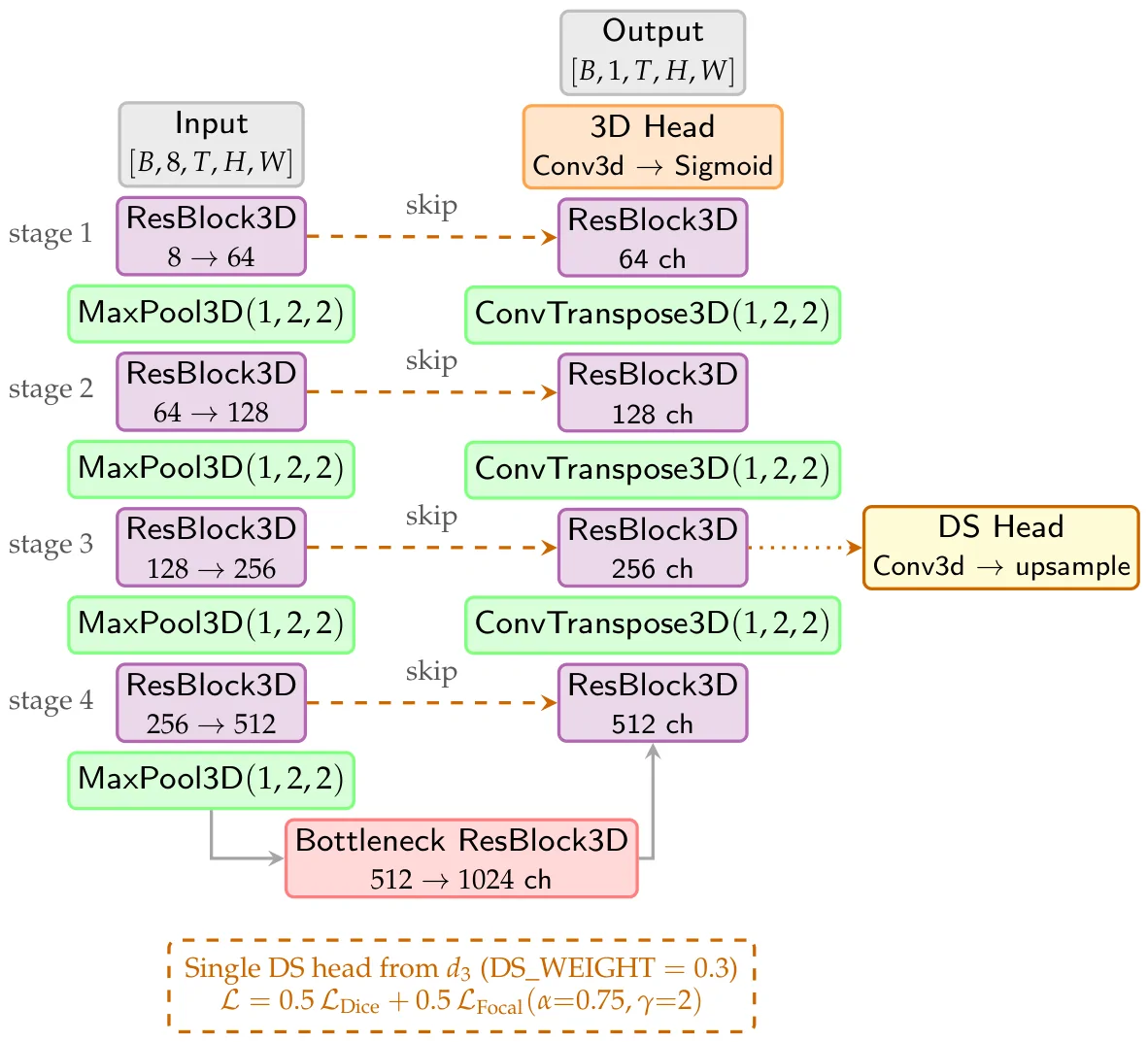
SpaSE-UNet3D AF variant. The encoder (**left**) takes an 8-channel VIIRS input and downsamples spatially through four ResBlock3D stages with channels [64,128,256,512]. A single ResBlock3D bottleneck widens to 1024 channels. The decoder (**right**) mirrors the encoder with channel concatenation at every skip. A 3D head produces per-day fire masks. Deep supervision is applied at the third decoder stage. Grey arrows show the forward data path, dashed orange arrows the encoder–decoder skips, and the dotted arrow the deep-supervision branch, whose weight and loss are given in the dashed box. Box fill marks the operation type only: violet, residual block; green, resampling; red, bottleneck; orange and yellow, output and deep-supervision heads; grey, tensors.

**Figure 3:**
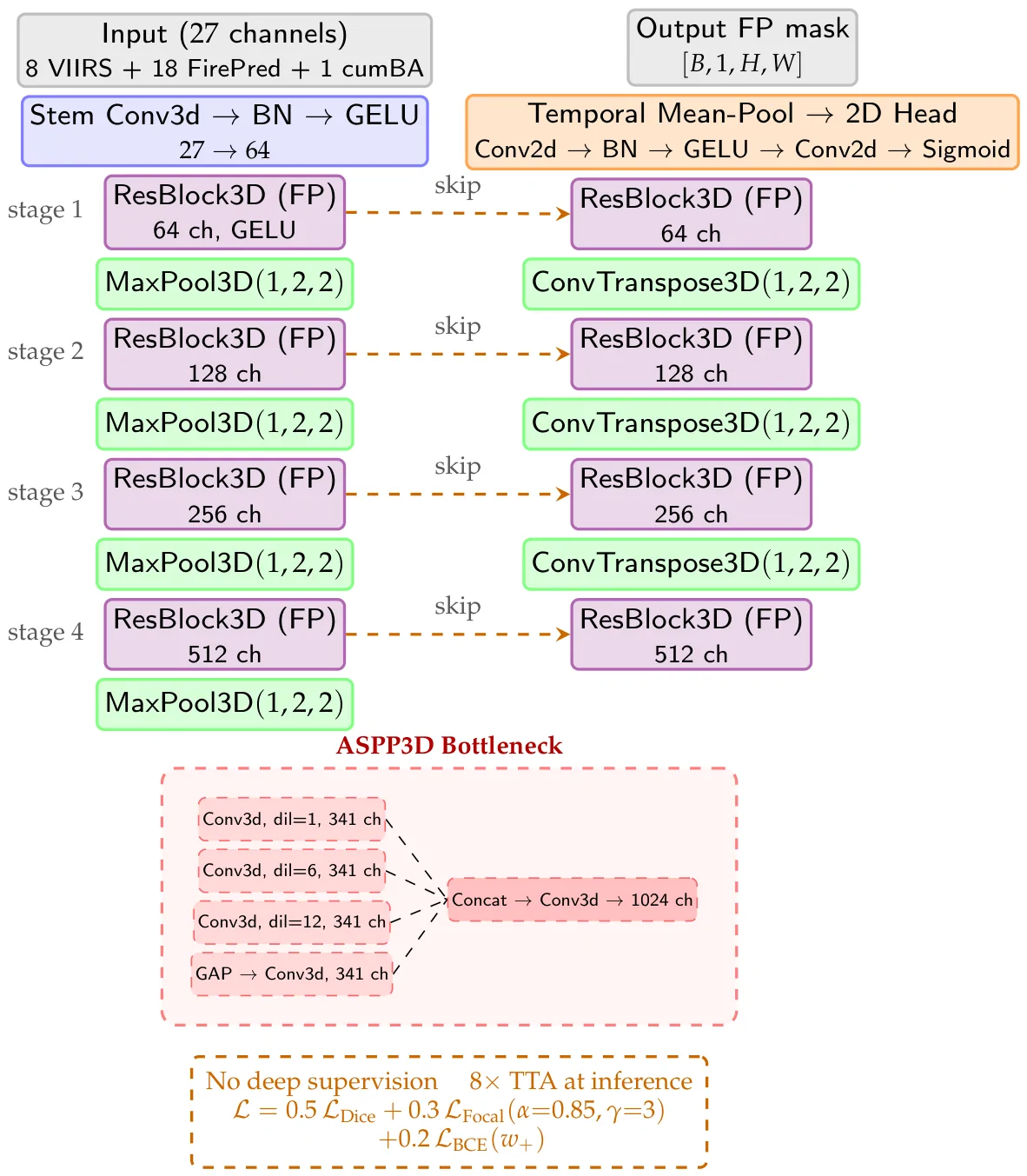
SpaSE-UNet3D FP variant. The encoder takes a 27-channel input combining VIIRS, FirePred auxiliary bands, and a cumulative burned-area mask. The bottleneck is replaced with an ASPP3D module that aggregates multi-scale context via parallel dilated convolutions and a global average pool. The final head collapses the temporal dimension via a mean-pool and applies a 2D convolutional head to produce a single-day FP mask. No deep supervision is used; 8× test-time augmentation is applied at inference. Dashed orange arrows are the encoder–decoder skips; the dashed red box encloses the four parallel ASPP3D branches, concatenated and fused along the dashed lines inside it; the lower dashed box gives the inference setting and the loss. Box fill marks the operation type only: blue, stem; violet, residual block; green, resampling; red, ASPP3D; orange, output head; grey, tensors. GAP: global average pooling; dil: dilation rate.

SpaSE-UNet3D is a family of two closely related encoder–decoder architectures, one for AF detection and one for FP prediction. Both variants share four design principles: (i) spatial-only (1,3,3) convolutions in every residual block, (ii) SE channel attention [13] inside every residual block, (iii) a symmetric encoder/decoder with skip concatenations as in U-Net [7,12], and (iv) a combined Dice + focal loss [27,28]. Task-specific variations handle the different input dimensionality, label density, and temporal-context budget of AF and FP. Figure 1 shows the shared residual block side-by-side; Figure 2 and Figure 3 show the AF and FP variants, respectively.

### 5.1. Shared Building Blocks

#### 5.1.1. Spatial-Only Convolutions

The central design choice of SpaSE-UNet3D is to extract features spatially and to combine the few available days only by global pooling, never by learned temporal filters. This is a deliberate departure from the isotropic 3D convolutions used by the U-Net-3D and transformer baselines, and it is motivated by the sensor regime: on windows of one or two daily acquisitions, there is little inter-day signal for a temporal filter to exploit, and the dataset paper’s own window-length ablation (Section 6.3) shows exactly this. Concretely, every feature-extraction convolution in the encoder and decoder residual blocks has kernel shape (1,3,3) with padding (0,1,1), so that no convolution in the network combines information across days. Other operations are not (1,3,3) convolutions: the residual skip paths use 1×1×1 projections, the decoder upsamples with ConvTranspose3D(1,2,2), the FP variant adds an input projection and an ASPP module with dilated branches, and the FP output head is two-dimensional. All of these are likewise spatial: none mixes across the temporal axis.

The temporal dimension *T* is therefore preserved through every encoder and decoder stage, and is never mixed *by convolution*. It is not, however, untouched: the SE modules pool globally over (T,H,W) when computing channel gates, and the FP head collapses *T* by a mean before its final 2D convolutions. The design claim is thus specific—temporal information is combined only by global pooling, never by learned temporal filters—and we state it in that precise form, not as an absence of temporal mixing altogether.

This design is validated in Section 6.5: the spatial-only network attains the same validation F1 as a full (3,3,3) variant while using 2.72× fewer parameters, confirming that on these short windows the temporal-mixing capacity of full 3D kernels is spent without return.

#### 5.1.2. SE Channel Attention

After the second convolution in every residual block, features pass through an SEBlock3D module with reduction ratio r=8. The module applies global average pooling over (T,H,W), a two-layer MLP with dimensions (ch,ch/r,ch), and a sigmoid to produce per-channel gating weights which multiply the input. This lets the network learn which VIIRS spectral bands or auxiliary features are informative per spatial context.

#### 5.1.3. Downsampling and Upsampling

Encoder stages are separated by MaxPool3D(1,2,2), which halves the spatial dimensions but leaves *T* unchanged. Decoder stages upsample with ConvTranspose3D(1,2,2), and the upsampled features are concatenated with the matching-resolution encoder skip.

#### 5.1.4. Residual Blocks

Each block applies two (1,3,3) convolutions with batch normalisation [29] and a non-linear activation, followed by SE attention and a residual addition with the (possibly projected) input. Both variants of the ResBlock3D are shown in Figure 1. The two variants differ only in the choice of activation (ReLU vs. GELU [30]) and the presence or absence of Dropout3D [31].

### 5.2. AF Variant

The AF variant takes an 8-channel input: the six daytime VIIRS bands and two nighttime VIIRS bands, stacked along the channel dimension of the temporal tensor. The encoder has four stages with output channels [64,128,256,512]. The bottleneck is a single ResBlock3D that widens to 1024 channels. The decoder mirrors the encoder with channels [512,256,128,64], each stage being a ResBlock3D operating on the concatenation of the upsampled feature map and the encoder skip. The full architecture is shown in Figure 2.

#### 5.2.1. ResBlock3D (AF)

The AF residual block (Figure 1, left) applies two (1,3,3) convolutions with batch normalisation, ReLU activation, Dropout3D (p=0.1) between them, an SE channel-attention module, and a residual addition with a projected (or identity) skip [32]. The Dropout3D inside the block regularises the network against the relatively small effective training set of 120 fires.

#### 5.2.2. Deep Supervision

To force intermediate decoder representations to be spatially specific, the AF variant attaches an auxiliary segmentation head to the third decoder stage ($d_{3}$, 256 channels), upsamples its output to the input resolution, and adds its loss to the total objective with weight DS_WEIGHT=0.3. Both the main and auxiliary losses are computed on the last temporal frame only: the model produces predictions for all *T* input days, but the AF labels are only available for the final day, so we supervise only that frame.

#### 5.2.3. Loss (AF)

The AF training objective is a weighted sum of Dice [27] and focal loss [28], with the deep-supervision term added at weight 0.3:

(3) $$L_{\mathrm{AF}}\;=\;0.5\;L_{\mathrm{Dice}}+0.5\;L_{\mathrm{Focal}}\left(\alpha \;=\;0.75,\gamma \;=\;2\right)+0.3\;L_{\mathrm{DS}}.$$

The Dice term directly optimises the F1 surrogate; the focal term handles the heavy class imbalance (fire pixels are typically <1% of the patch).

### 5.3. FP Variant

The FP variant takes a 27-channel input: the 8 VIIRS bands, 18 FirePred auxiliary bands (band 3, total precipitation, is excluded), and 1 cumulative burned-area mask computed from all days up to *T*. The wider probe reported in Section 3 shows this band to be uniformly zero in 12 of 62 fires and informative in the remainder, so including it is a natural extension of the present configuration. The encoder follows the same four-stage schedule [64,128,256,512], but is preceded by a dedicated input-projection layer Conv3d(27,64,(1,3,3))→BN→GELU that mixes the heterogeneous input channels before the first encoder block. The full architecture is shown in Figure 3.

#### 5.3.1. ResBlock3D (FP)

The FP residual block (Figure 1, right) differs from the AF block in two ways. First, GELU [30] replaces ReLU as the activation; empirically, we found that the sparser loss landscape of the FP objective benefits from the smoother gradient of GELU. Second, there is no Dropout3D [31] inside the block: on the much smaller effective FP training set (89 fires, with sparse window-level positives), Dropout hurt convergence. The skip connection also drops the BatchNorm on the projection to keep the residual path closer to identity.

#### 5.3.2. ASPP3D Bottleneck

The FP variant replaces the plain bottleneck of the AF variant with a 3D Atrous Spatial Pyramid Pooling module [14,15]. Four parallel branches operate on the encoder output at stride 16:

- Conv3d(512,341,(1,3,3)) with spatial dilation rate 1;
- the same with spatial dilation rate 6;
- the same with spatial dilation rate 12;
- global average pooling over (H,W) (preserving the temporal dimension *T*) followed by Conv3d(512,341,1) and broadcast back to the spatial grid.

The four branch outputs (each 341 channels) are concatenated (for 4 × 341 = 1364 channels) and fused with a Conv3d(1364,1024,1)→BN→GELU. The dilated branches capture context at multiple spatial scales, which is critical because fire-front widths in the TS-SatFire test set range from a few newly burned pixels to hundreds.

#### 5.3.3. Final Head

The FP head first collapses the temporal dimension with a mean over *T*, then applies a 2D head Conv2d(64,32,3)→BN2d→GELU→Conv2d(32,1,1)→Sigmoid. The collapse is appropriate because the FP label itself is 2D (a single-day progression mask), while the AF head remains 3D because AF labels are per-day.

#### 5.3.4. No Deep Supervision

We tested deep supervision for FP and removed it. The sparse, imbalanced FP labels combined with the small effective training set caused auxiliary losses to introduce conflicting gradients that hurt convergence.

#### 5.3.5. Loss (FP)

The FP training objective extends the AF loss with a weighted binary-cross-entropy term:

(4) $$\begin{array}{c} L_{\mathrm{FP}}=0.5\;L_{\mathrm{Dice}}+0.3\;L_{\mathrm{Focal}}\left(\alpha \;=\;0.85,\gamma \;=\;3\right)\\ \;+0.2\;L_{\mathrm{BCE}}\left(w_{+}\right). \end{array}$$

The addition of BCE is necessary because the FP target is far sparser than AF: typical windows have <0.05% positive pixels. The positive weight $w_{+}$ is not a hand-tuned constant: it is computed at preload time from the observed positive-pixel ratio of the training split, and therefore varies slightly between runs (116.6–117.3 across our runs). This removes one hyperparameter from the design and ties the term directly to the class balance it is intended to correct. The higher focal γ=3 (vs. γ=2 in AF) further downweights easy negatives.

#### 5.3.6. Test-Time Augmentation

At inference, the FP variant uses 8× test-time augmentation [33]: the input is passed through horizontal flip, vertical flip, both, and three 90° rotations, plus the identity, and the resulting logits are averaged before the sigmoid and threshold.

## 6. Experiments

### 6.1. Implementation Details

All experiments were conducted on a single NVIDIA T4 GPU (16 GB VRAM) using PyTorch 2.10 with automatic mixed precision (AMP). A single training run takes between 3.5 and 6.5 h depending on task and window length; one run of each of the three configurations completes in approximately 14 h.

#### 6.1.1. Shared Hyperparameters

Batch size 8; patch size 256×256 (centre crop of the full scene); optimiser AdamW [34,35] with learning rate $5\times 10^{-4}$ and weight decay $1\times 10^{-4}$; OneCycleLR [36] schedule with pct_start=0.1 and cosine annealing; gradient clipping at norm 1.0. Each of the three configurations—AF at TS = 1, AF at TS = 2, and FP at TS = 2—is trained with five independent random seeds. Every figure we report is a mean over those runs with the standard deviation given alongside, and the per-seed outputs are released with the code.

#### 6.1.2. AF

80 epochs for TS = 2, 100 epochs for TS = 1. VIIRS channels are normalised with per-band means and standard deviations computed once on a probe fire and reused across runs.

#### 6.1.3. FP

60 epochs. VIIRS channels use the same AF normalisation; FirePred auxiliary bands are normalised with per-band statistics computed at preload time on the training split and cached so that validation and test evaluation use identical statistics. Training data is augmented with horizontal and vertical flips plus 90° rotations; test-time augmentation is applied at inference as described in Section 5.

#### 6.1.4. Sampling

For AF, we keep only training windows that contain at least 10 fire pixels and include at most 2 times as many negative windows as positive. For FP, we use at least 3 burned pixels as the positive threshold and a negative-to-positive ratio of 1.5. These heuristics stabilise training without overfitting to the positive class.

#### 6.1.5. Threshold Selection

For both tasks, we sweep the inference threshold over the validation set and select the value that maximises validation F1, independently in each run, then apply it unmodified at test time. No test-set information enters the selection. The selected AF value varies widely between runs while test F1 does not, which we report as evidence of insensitivity in Section 6.3; the FP runs selected the same value, 0.75, throughout.

#### 6.1.6. Metrics

For all tasks, we report pixel-level F1 and IoU (Jaccard index) aggregated over all pixels in all test fires (micro-average). Precision and recall at the selected operating point, and per-fire F1 and IoU, are also reported. Section 6.2 states the full evaluation protocol and its relationship to the reference implementation.

### 6.2. Evaluation Protocol

All baseline figures in this paper are quoted from Zhao et al. [5]; we did not retrain or re-evaluate them. Our own evaluation was implemented independently, and both works measure the same two tasks. The scoring procedures differ, however, and this section documents how, so that the figures we report are interpreted correctly.

The difference is visible in the reported metrics themselves. For metrics accumulated over a pixel population, F1 and the Jaccard index are related by a deterministic identity,

(5) $$\mathrm{IoU}=\frac{F1}{2-F1},$$

which our own measurements satisfy to four decimal places. The published pairs are related differently—SwinUNETR-3D at TS = 2 reports F1 = 0.823 with IoU = 0.727, where the identity would give 0.699—which indicates that the two works aggregate per-pixel counts differently: averaging a score computed per frame and per fire, rather than accumulating counts and computing the metric once. Both are legitimate choices, but they yield different quantities, and figures computed under one should not be compared directly with figures computed under the other.

Reading the reference implementation identifies eight further differences (Table 3). Four are stated in the dataset publication [5]—the two label rules, the replacement of missing values with zeros, and the test-set sampling interval—and are choices we depart from rather than omissions. The remaining four are recoverable only from the released code, and two of those bear directly on the reported figures: the aggregation described above, and the convention that frames with empty ground truth are scored 1.0 rather than omitted, which affects the two test fires carrying no active-fire annotation (Section 4). We note these not as errors but because a reader cannot reproduce the published figures without them.

Because these differences are separable, their cost can be measured. Table 4 applies them cumulatively to our AF model with the weights held fixed, so that only the measurement changes. The decomposition is carried out on the reference implementation’s 256×256 crop, placed at a 30% offset from the scene origin rather than centred, so that the spatial extent scored is the same in every row. The first row therefore reports our active-fire label rule and our final-frame scoring measured on that crop, and each subsequent row adds one further reference convention. Because the crop differs from the centred one used throughout the rest of this paper, the first row is not the configuration of Table 5 and should not be read against it; the quantity of interest here is the spread down the column rather than any single entry.

The scoring convention thus moves F1 by 0.10 on fixed weights, a range comparable to that separating the twelve published baselines (0.713–0.823). Our figures and the published ones are therefore not placed on a single axis. Measured under our protocol, our results reach or surpass the level of the strongest published baselines on both tasks, and they do so from a two-day observation window rather than six—an efficiency margin that holds regardless of scoring convention.

The interaction between the first two rows of Table 3 accounts for much of this gap, and is worth stating explicitly because it is easy to overlook. The reference evaluation scores each day of each fire separately, using the instantaneous rather than the accumulated active-fire label, and awards a score of 1.0 whenever the reference mask for that day is empty and the model predicts nothing. Empty days are common in this benchmark: they occur before ignition, after burnout, and on days when cloud cover prevents detection. Every such day contributes a perfect score to the average, so the reported figure combines segmentation quality on days with fire and correct abstention on days without. A micro-averaged figure contains no equivalent term, since days without positive pixels contribute no counts. The two conventions measure different quantities, and the difference is not recoverable from the published tables alone.

The decomposition of Table 4 isolates the scoring effect precisely because the model is held fixed: any movement in the reported number is attributable to the measurement alone. Retraining the baseline architectures instead would introduce a second source of difference—the reimplementation’s hyperparameters, convergence behaviour and training budget, none of which are documented in enough detail in [5] to reproduce exactly—so a gap between a retrained baseline and its published figure could not be attributed to either cause. The two approaches answer different questions, and retraining all baselines under a single stated protocol is the right way to establish how the architectures rank under a common evaluation.

All results below therefore use the following: counts accumulated over all pixels with the metric computed once; unannotated fires excluded and reported separately rather than scored; AF labels as band 7≥7 on the final day of each window; FP targets as the clipped increment between consecutive burned-area masks; a centred 256×256 crop; and a threshold selected on validation and applied unmodified at test time. Per-seed outputs are released with the code.

### 6.3. Active Fire Results

Table 5 reports our test-set F1 and IoU at TS = 1 and TS = 2 alongside the twelve baselines of the dataset paper [5]. The two groups are listed separately because they are scored differently (Section 6.2): the baseline figures are reproduced as published, while ours are micro-averaged over the 15 annotated test fires at a validation-selected threshold. The table places the published landscape next to an independently measured result; it is not a ranking.

Under our protocol, SpaSE-UNet3D reaches F1 = 0.8549±0.0005 at TS = 2 and F1 = 0.8520±0.0008 at TS = 1, each averaged over five independent runs. The difference between the two is small, and of the same order as the spread between repeated runs of the same configuration, so we treat the one-day and two-day results as substantially equivalent rather than ordering them. Per-fire inspection shows this equivalence is not uniform: individual fires shift by up to 0.022 in either direction between TS = 1 and TS = 2, with the largest losses on the weakest-signal events (Swedish, Blue Ridge). The second day therefore appears to add and remove information in roughly equal measure, which is consistent with there being little fire-specific signal in the additional frame. The same tendency appears in the dataset paper’s own ablation on window length, where SwinUNETR-3D improves monotonically on active fire as the window shortens (0.783 at TS = 6, 0.803 at TS = 4, 0.823 at TS = 2), which the authors attribute to the additional temporal information not being effectively used [5]. Our architecture is a direct response to that observation: if the extra frames are not exploited, the capacity allocated to mixing them is better spent elsewhere.

These figures are worth reading in absolute terms, independently of any comparison. An F1 of 0.8549±0.0005 at TS = 2 corresponds to a precision of approximately 0.85 and a recall of approximately 0.86 on the audited test fires: of the pixels the model flags as active fire, roughly six in seven are correct, and of the truly burning pixels it recovers a similar fraction. The two are balanced fire by fire as well as in aggregate—per-fire precision spans 0.740–0.921 and per-fire recall 0.670–0.895, with neither systematically above the other—so the aggregate does not conceal a precision-favouring or recall-favouring operating point. On a 375 m sensor grid this is an operationally useful level of agreement with verified ground truth, and it is reached from a two-day observation window and, within 0.003 F1, from a single day. We regard the stability of the result as part of its quality: across five independent seeds, the standard deviation is 0.0005 F1, so the number is a reproducible property of the model and not of a fortunate initialisation—and it is more than an order of magnitude tighter than the spread the same pipeline produces on fire progression, where the data are far sparser.

Threshold selection is performed independently in each run by sweeping the validation split and retaining the value that maximises validation F1, which is then applied unmodified at test time. The selected values differ substantially between runs while test F1 does not, which is itself the relevant result: performance is essentially flat in the threshold. A sweep over the test set confirms this, with F1 varying by less than 0.005 across the entire range [0.20,0.80] as precision and recall trade off symmetrically. In every run, the validation-selected threshold yielded test F1 within 0.0003 of the best achievable on the test set, so no advantage accrues from the selection procedure; we report the test sweep as evidence of robustness only, and it plays no part in selection.

**Figure 4:**
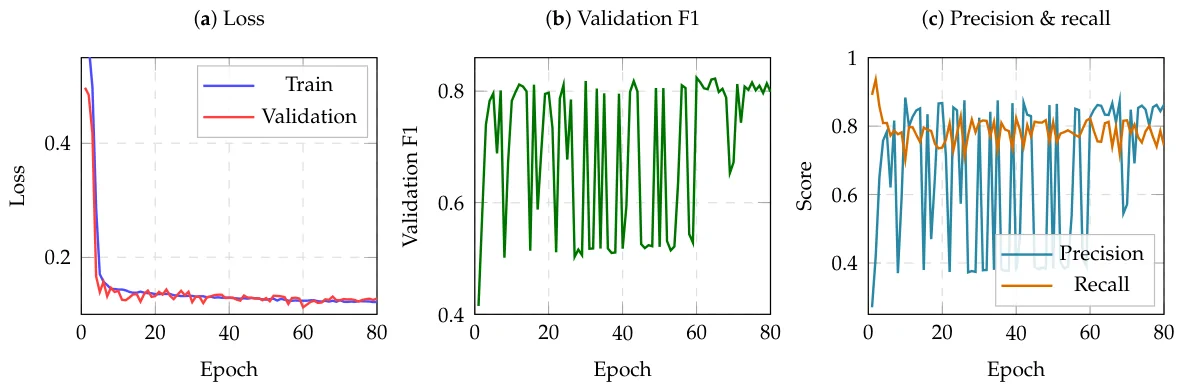
Training behaviour of the AF variant at TS = 2 for a representative run. (**a**) Training and validation loss remain closely coupled. (**b**) Validation F1 rises above 0.80 within ten epochs and then oscillates as the operating point moves. (**c**) Validation precision and recall: recall is the binding constraint for much of training, which is the behaviour the label audit of Section 4 addresses.

Training behaviour is shown in Figure 4. Validation F1 rises above 0.80 within the first ten epochs and then oscillates as the operating point shifts between precision- and recall-favouring regimes, stabilising as the OneCycle schedule anneals the learning rate. Training and validation loss remain closely coupled throughout, with no divergence indicative of overfitting. Figure 4c is the empirical basis for the audit of Section 4: recall sits near 0.80 for much of training while precision oscillates widely, the asymmetry expected when some training windows are supervised by all-negative targets derived from unannotated scenes.

**Figure 5:**
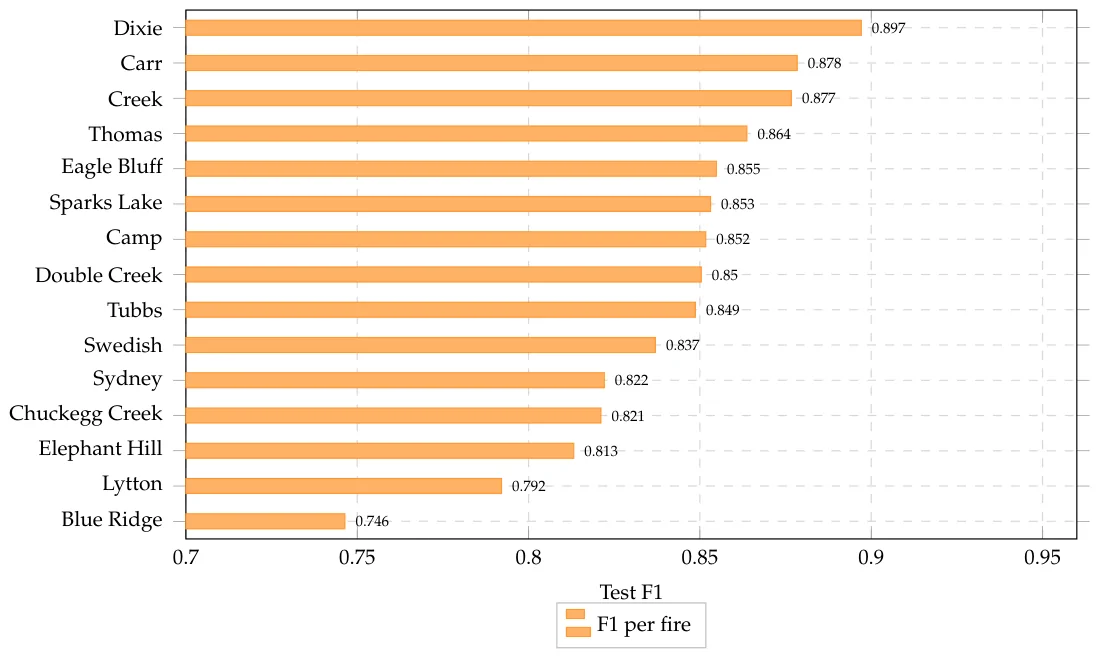
Per-fire test F1 for SpaSE-UNet3D at TS = 2 on the 15 annotated AF test fires, sorted by F1. Measured under the protocol of Section 6.2.

The aggregate test F1 is informative but masks per-fire variability. Figure 5 breaks down test F1 across the 15 annotated test fires. Moreover, 14 of 15 fires reach F1 ≥0.792, and the median fire (*Double Creek*) reaches F1 = 0.850. Eight fires exceed F1 = 0.85. *Double Creek* warrants a note: it carries active-fire annotation on only 3 of its 10 days, and 70.3% of its labelled positive pixels fall outside the centre crop (Section 3.3). Because it is scored only on its annotated days, its inclusion introduces no unlabelled-data bias, and removing it entirely changes the aggregate F1 by less than $10^{-4}$, so it is retained. The single outlier is the low-intensity *Blue Ridge* fire at F1 = 0.746; manual inspection of its VIIRS scenes shows that the active-fire signal is faint, often confined to a single pixel, and consistently obscured by smoke plumes that drift from neighbouring confirmed-fire pixels.

**Figure 6:**
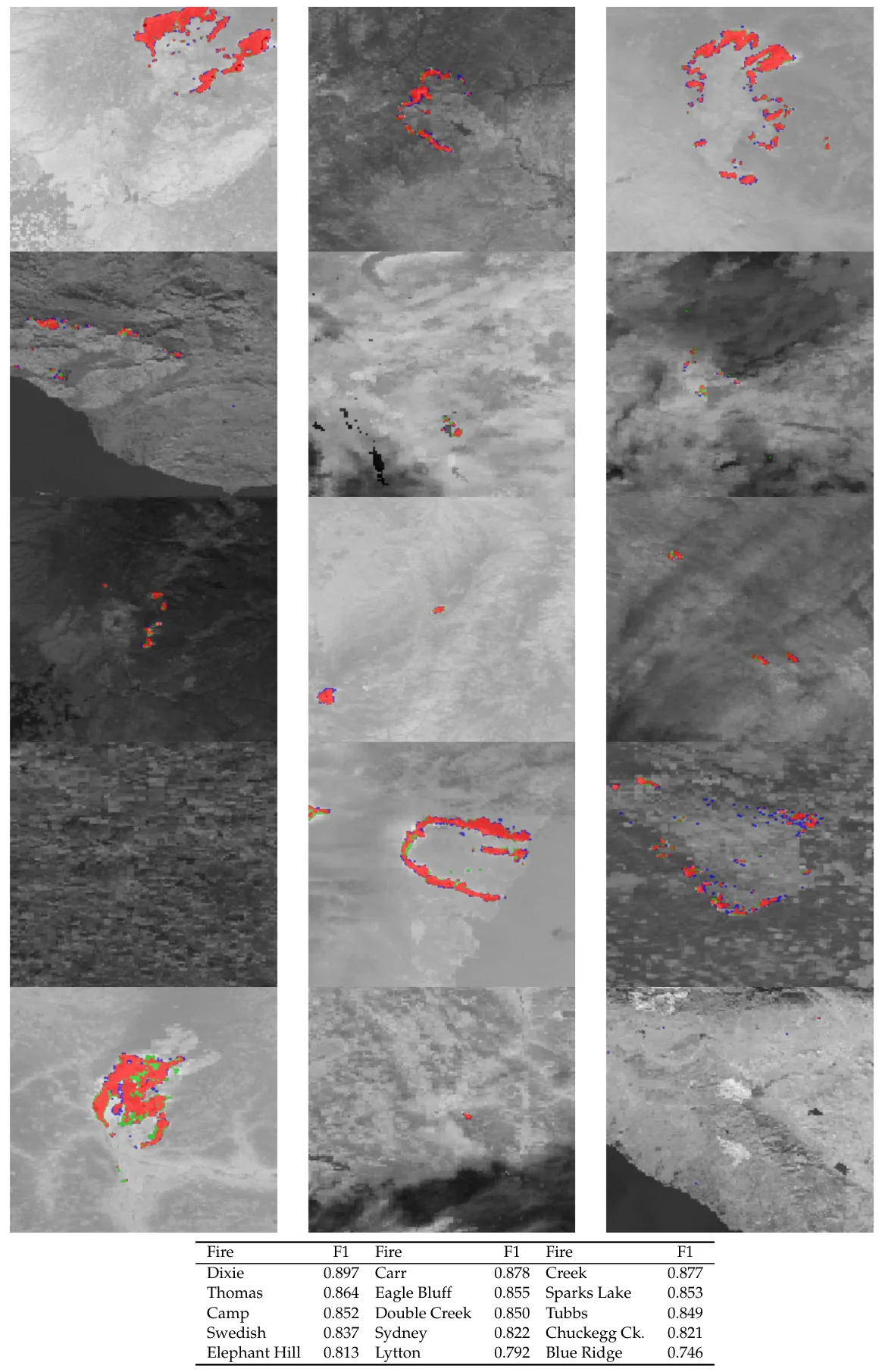
AF test-set predictions (TS = 2) for the 15 audited test fires, sorted by F1 from top-left to bottom-right. Each panel overlays the per-pixel confusion mask on the VIIRS day scene: red = true positive, green = false positive, blue = false negative. Per-fire F1 scores are listed in the table above.

To verify that the per-fire numbers reflect spatially coherent predictions and not artefacts of the aggregate F1, Figure 6 shows true-positive (red), false-positive (green), and false-negative (blue) overlays for all 15 test fires at the optimal threshold. The dominant red colour across all panels confirms that the bulk of pixel-level predictions agree with the audited ground truth. False positives are typically small clusters along smoke or hot-soil boundaries (*Sydney*, *Chuckegg Creek*), and false negatives concentrate on the periphery of large active fronts (*Dixie*, *Creek*) where the radiometric signal is borderline. Crucially, no fire shows a systematic spatial bias, which is consistent with the symmetric precision/recall behaviour reported above.

#### Impact of the Audit

To isolate the effect of label filtering, we trained two models differing in exactly one respect—whether windows drawn from unannotated days are retained with all-negative targets—with all other settings held fixed. This is a single-run controlled pair, separate from the five-seed configuration of Table 5, and its two figures should be read against each other rather than against the headline mean. The audited model reaches test F1 = 0.8533 against 0.8514 for the unaudited one, a difference of 0.0019. The effect is small because day-level filtering (Section 4) already rejects any window whose final day lacks a label, so only 71 of 1790 windows differ between the two conditions. The practical implication is that day-level verification does most of the work, and that the failure mode appears at full magnitude only in a pipeline with neither filter. The value of the audit lies elsewhere: two of the seventeen AF test fires carry no annotation and cannot be scored, so any full-split aggregate depends for those fires on the empty-ground-truth convention rather than on the model.

### 6.4. Fire Progression Results

Fire progression is evaluated on the full official test set of 24 US_2021 fires. Eight of these carry little or no measurable progression signal inside the crop, and reporting them alongside the rest costs 0.033 F1 and—consistent with the crop analysis of Section 3.3—raises the run-to-run standard deviation from 0.012 to 0.022. We report both populations in Table 6 so that the effect of these fires on the aggregate is visible rather than absorbed into it.

Table 6 reports our result alongside the five published FP baselines. As in Section 6.2, the two groups are listed separately because they are scored differently: the baselines are reproduced as published under the reference protocol, and ours is measured under the protocol of Section 6.2. On the 16 fires where the crop retains a measurable progression signal, our figure stands 0.043 above the highest value reported for a published baseline—more than three times the run-to-run standard deviation of that population—while over the full 24-fire set the separation narrows to 0.010 and falls within seed noise. Both readings are measured on our own terms, and the difference between them is a property of the test population rather than of the model.

Run-to-run variance is worth reporting alongside the aggregate, and is informative in a way we did not anticipate. Across five runs differing only in random seed, our test F1 on the full test set has a standard deviation of 0.0221. Zhao et al. [5] also report a seed study on this task, over comparable seeds, and obtain a standard deviation of 0.003—several times smaller. Since both studies vary only the random seed, the difference is attributable to how per-pixel counts are turned into a reported score: averaging a metric computed per frame and per fire concentrates the distribution, whereas accumulating counts across the whole test set does not. Aggregation therefore affects the dispersion of a reported figure as well as its value, which is a further reason to read the two sets of numbers separately.

Under our own aggregation, the seed noise (0.0221) is essentially equal to the 0.021 range separating the five published fire-progression baselines from one another, so differences of that size are not reliably resolved by a single run measured this way. This applies to our own results first of all, which is why every figure we report on this task is a mean over five seeds with its standard deviation given alongside.

Instability is visible during training as well. The epoch of best validation F1 varied by more than twenty epochs across runs while validation F1 at that epoch varied far less than test F1 did, indicating that validation performance is a weak predictor of test performance on this task. With only eight validation fires carrying usable progression windows (Section 4), this is expected, and it is a structural limitation of the benchmark rather than of any particular model. The contrast with active fire detection, where the same architecture and pipeline give a far smaller spread, points to label sparsity and the small number of independent fire events—not the model—as the source.

The threshold was selected on the validation split in every run and applied unmodified at test time; all five runs selected the same value, 0.75. Validation F1 and IoU rise gently and monotonically across the swept range [0.05,0.75] with no sharp peak, so the choice is not critical.

**Figure 7:**
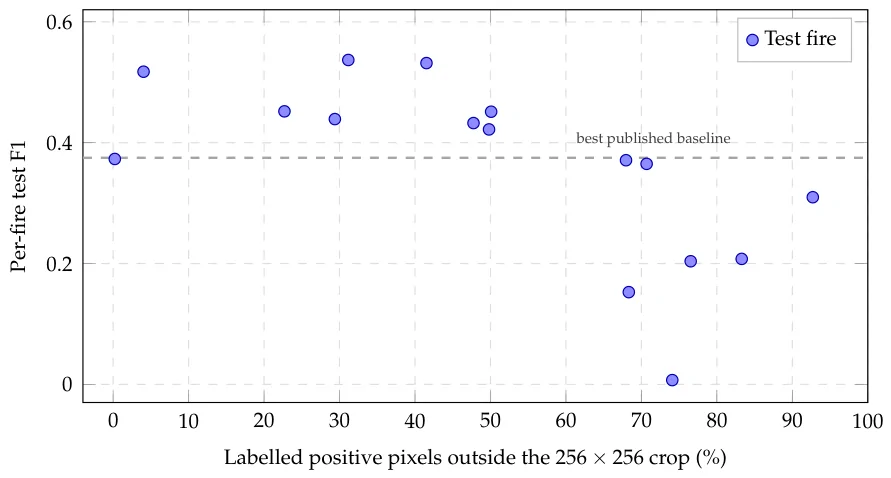
Per-fire fire-progression F1 against the fraction of labelled positive pixels falling outside the 256×256 centre crop (Section 3.3), over the 16 test fires for which per-fire diagnostics were exported (Figure 8); the remaining fires are scored in the aggregate of Table 6 but were not written out individually. Fires whose extent is largely cropped away score poorly regardless of model behaviour; Spearman ρ=−0.685, p=0.0034, n=16. Each blue circle is one test fire; the dashed grey line marks the F1 of the best published baseline, 0.375.

**Figure 8:**
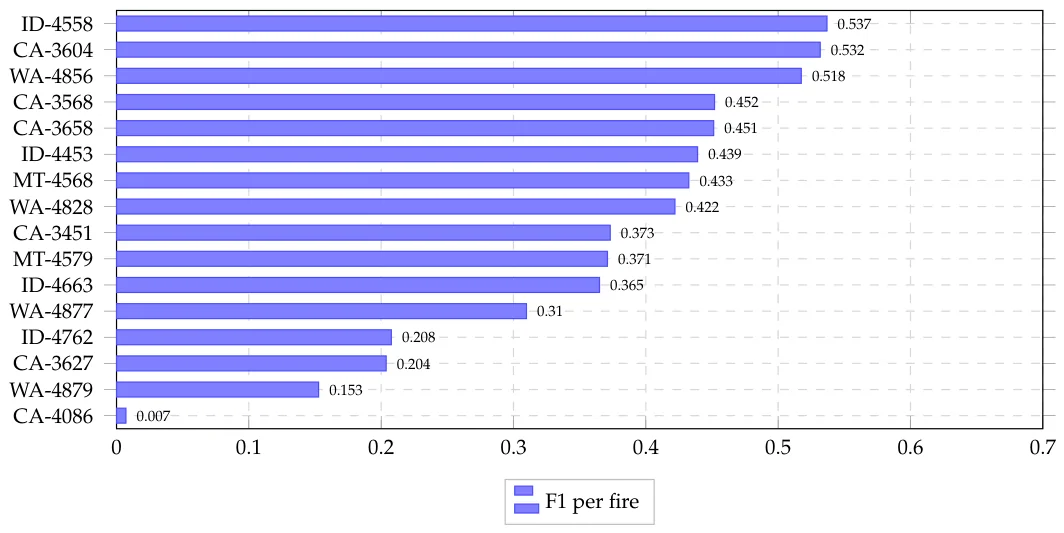
Per-fire test F1 for SpaSE-UNet3D on 16 of the US_2021 test fires from one representative run, sorted by F1. The chart is shown to illustrate the spread between events rather than to reproduce an aggregate: the headline figures in Table 6 are means over five runs on all 24 official test fires, of which six carry no usable progression window and therefore contribute no counts.

Aggregate performance conceals wide per-fire variation: per-fire F1 ranges from below 0.01 to above 0.53 in a representative run, and that spread is the feature of interest rather than any individual value. Measurement identifies a concrete and sufficient cause for the low-scoring cases: truncation of the fire by the 256×256 crop. Across the progression test set, a mean of 56.3% of labelled positive pixels fall outside the crop (Section 3.3). Per-fire F1 correlates strongly and negatively with the fraction of positive pixels lost to the crop (Figure 7; Spearman ρ=−0.685, p=0.0034). Fires scoring below 0.375 lose, on average, 66.7% of their positive pixels to the crop; those scoring above lose 34.6%. A model asked to predict progression on a crop containing a small fragment of the fire front is being evaluated on a task whose ground truth is largely absent from its input. The extreme case, *CA-4086* at F1 = 0.007, has eight usable windows in total with a progression increment occupying less than 0.01% of the crop; the model returns near-zero probability across the scene, which is the correct response to the input it is given—and is scored close to zero for it. The per-fire breakdown of the same run, over 16 of the test fires, is shown in Figure 8.

Centre-cropping is a standard convention adopted for memory reasons, but on this dataset it discards the majority of the progression signal, so per-fire scores partly reflect how much of each fire survives the crop. Full-scene evaluation is left to future work.

**Figure 9:**
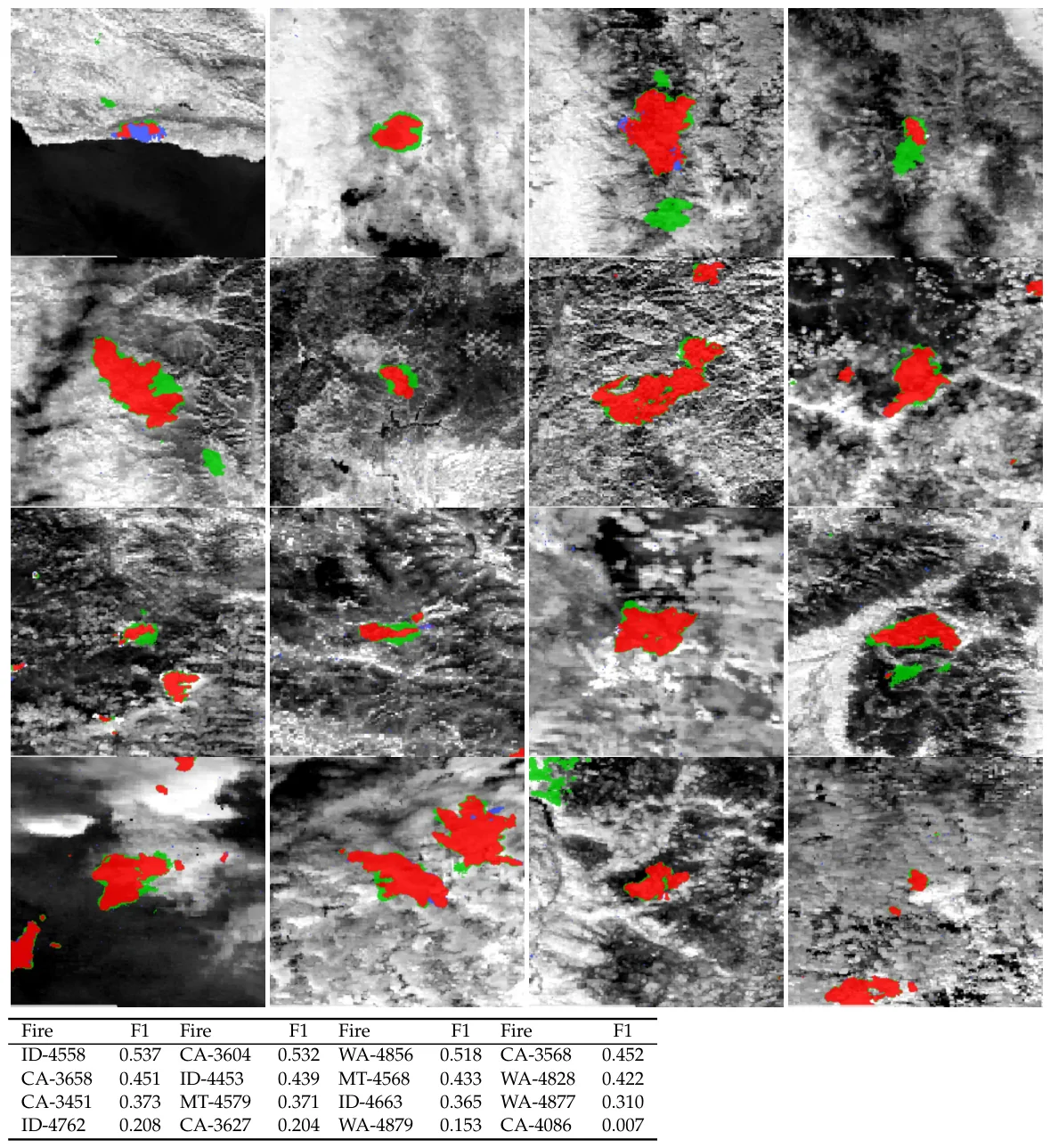
Qualitative fire-progression predictions for 16 US_2021 test fires from a single run, sorted by F1 from top-left to bottom-right. The panels are illustrative of the model’s spatial behaviour and are not the population over which the reported metrics are computed; those are given in Table 6 over all 24 official test fires. Each panel overlays the predicted progression mask on the VIIRS day scene: red = true positive, green = false positive, blue = false negative. Per-fire F1 scores are listed in the table above.

To examine prediction quality qualitatively, Figure 9 shows one representative window per fire for 16 test fires from a single run, ordered by F1 from best (top-left) to worst (bottom-right). Most fires display predicted progression masks that closely follow the ground-truth perimeter shape: the model propagates the active-fire front in the direction implied by the cumulative burned-area channel and the FirePred terrain and weather covariates. Misalignment, where it occurs, manifests as a slight directional offset (*ID-4558*, *CA-3658*) instead of a complete shape mismatch. The lower-F1 fires reveal the dominant failure mode identified above: *ID-4762*, *CA-3627*, and *WA-4879* have very small ground-truth progression footprints relative to the crop, so that a single mispredicted patch dominates the score. These cases motivate reporting per-fire F1 alongside the aggregate, since a single truncated fire can shift the micro-averaged figure disproportionately.

Table 7 summarises the resource footprint for both tasks. SpaSE-UNet3D’s parameter counts—24.28 M for FP and 32.88 M for AF—sit in the same regime as the published 3D U-Net and transformer baselines. Its efficiency advantage lies in the two quantities that are directly comparable across protocols: the observation window, two days against the baselines’ six, and the training cost, between 3.5 and 6.5 h per configuration on a single NVIDIA T4.

### 6.5. Component Ablation

Table 8 reports four configurations, trained to isolate the contribution of the individual design choices: the full model, a variant replacing every spatial-only (1,3,3) kernel with a full (3,3,3) kernel, a variant with the squeeze-and-excitation modules removed, and a variant without the deep-supervision head. All arms used an identical 40-epoch budget and identical data, sampling, optimiser and schedule, and were evaluated on the validation split so that no test-set information entered any design decision. The full configuration was trained three times to estimate run-to-run variability; the three reduced configurations were trained once each.

The four configurations agree closely: run-to-run standard deviation on the full model is σ=0.0009, and every reduced variant lies within 1.6σ of it. At this sample size, the arms are statistically indistinguishable in accuracy, and we make no claim that any individual component improves it. Accuracy on this task is therefore not governed by any one of these design choices—a robustness result in its own right, and one that bears on how the individual components should be justified. The squeeze-and-excitation modules are retained because they supply their channel reweighting for 0.44 M parameters, 1.3% of the model, at no measured cost in accuracy; the deep-supervision head shows the largest single deviation (1.6σ), and its contribution is the one this measurement comes closest to resolving.

The ablation does, however, establish a clear and useful result about efficiency. The spatial-only design attains the same validation F1 as the full (3,3,3) alternative—the two agree to within 1.1σ—while using 2.72× fewer parameters (32.88 M against 89.36 M). On the one- to two-day windows this benchmark provides, in other words, the model recovers the full accuracy of isotropic 3D convolution at a third of its parameter cost: the temporal-mixing capacity that full kernels add is simply not needed at this window length. The squeeze-and-excitation modules deliver their channel reweighting for a further 0.44 M parameters, 1.3% of the model. This is the parameter-efficiency behaviour reported for factorised spatiotemporal convolutions in the action-recognition literature [10,11], and to our knowledge this is its first demonstration in the wildfire-segmentation setting.

We did not ablate the fire-progression variant: the run-to-run standard deviation of 0.0221 on that task (Section 6.4) is far larger than any plausible single-component effect, so a single-run ablation would be uninterpretable.

The provenance of the FP design choices is as follows. The variant reached its present form through six successive revisions, each evaluated once on the validation split of eight usable fires, with the loss weights (0.5 Dice, 0.3 focal, 0.2 weighted BCE), the focal parameters and the sampling ratios set by hand; the BCE positive weight is derived from the class balance of the training split at preload time rather than chosen. Selection was therefore sequential rather than exhaustive, and the configuration is the one we settled on for this data regime. A systematic search over that space—including the cumulative burned-area input channel, which supplies information that some baselines may not receive—is identified as future work in Section 8.

## 7. Discussion

SpaSE-UNet3D is, to our knowledge, the first fully spatial-only 3D architecture proposed for wildfire segmentation. It attains the accuracy of an isotropic (3,3,3) network with 2.72× fewer parameters (Section 6.5), performs comparably with a single day’s acquisition as with two (Section 6.3), and trains both tasks in under 15 h on one GPU. The parameter result is one of efficiency, since that is what the ablation measures directly. Establishing these claims required work that became a contribution in its own right: a model cannot be evaluated on a benchmark whose scoring is undocumented and whose labels are in part absent, and evaluating ours revealed both to be true of TS-SatFire. The audit, protocol decomposition and exclusion lists are therefore not a digression but the conditions under which the architecture—or any model—can be fairly assessed on this data. Three claims follow.

### 7.1. Scoring Convention Affects Reported Figures as Much as Architecture Does

Scoring a fixed set of weights under the reference procedure moves F1 by 0.10 (Section 6.2), as much as separates the twelve published baselines from one another. Figures produced under different conventions are therefore not on a common axis, and the identity IoU=F1/(2−F1) offers a one-line check of whether a published pair is micro-averaged before it is compared with one that is.

### 7.2. Run-to-Run Variance on Fire Progression Is Large Relative to the Differences Being Compared

Seeds differing in nothing else give a standard deviation of 0.0221 on the progression test set, essentially equal to the 0.021 range separating the five published baselines. Single-run comparisons on this task therefore cannot reliably distinguish architectures, our own included. Active fire detection behaves differently under the same pipeline, with a spread more than an order of magnitude smaller; the difference lies in label sparsity and in the small number of independent fire events, not in the models. Reporting seed variance before attributing a difference to an architecture seems to us the more transferable lesson of this study.

### 7.3. Spatial-Only Convolutions Suit the Short-Window Regime

On one- to two-day windows, the spatial-only design matches full 3D accuracy at a third of the parameters (Section 6.5), and the near-equality of our TS = 1 and TS = 2 results points the same way: there is little inter-day signal for a temporal filter to exploit. The design assumes only a short stack of co-registered multispectral rasters and a per-pixel target, so it should transfer to Sentinel-2, Sentinel-3 SLSTR or MODIS stacks, though we have not tested this.

### 7.4. Limitations

Our conclusions rest on a single benchmark and sensor family. The baselines were not retrained under our protocol, so the decomposition of Section 6.2 isolates the measurement effect on our own weights but leaves open how the baseline architectures would rank under a common evaluation. The ablation uses one run per arm and rules out only large effects; the fire-progression variant, given its seed variance, is not ablated. We train no burned-area model, leaving open whether the band-8 Julian-day codes can support multi-class or regression targets.

## 8. Conclusions

We presented SpaSE-UNet3D, a family of spatial-only 3D U-Nets with Squeeze-and-Excitation channel attention, together with the first systematic label-quality and evaluation-protocol audit of the TS-SatFire benchmark. SpaSE-UNet3D is a compact, spatial-only design for this sensor regime: it attains the accuracy of a full 3D network with 2.72× fewer parameters, performs comparably with one day’s acquisition as with two, and reaches F1 = 0.8549±0.0005 on active fire detection and F1 = 0.3845±0.0221 on fire progression under the audited protocol of Section 6, training both tasks in under 15 h on a single NVIDIA T4 GPU. Evaluating it fairly required first establishing that the benchmark’s published figures rest on a scoring convention and label set that differ from an independently implemented one, and that two of the seventeen active-fire test fires carry no annotation and cannot be scored at all. This audit and protocol analysis is a contribution in its own right: we release the exclusion lists, the protocol specification, and the per-seed outputs so that future comparisons on this benchmark can be made on a stated and common basis. On their respective terms, our figures match or exceed the strongest published baselines on both tasks, and SpaSE-UNet3D reaches this level of detection from a two-day observation window against the baselines’ six—an advantage in observation cost that is directly comparable across conventions.

Four directions follow directly from the limitations of this study. First, a properly powered ablation of the fire-progression variant, which the measured run-to-run standard deviation of 0.0221 places beyond the reach of single-run experiments. Second, retraining the baseline architectures under a single stated protocol, which would isolate architectural differences from measurement conventions. Third, full-scene rather than centre-cropped evaluation, since a mean of 56.3% of progression-labelled pixels fall outside the 256×256 crop and crop truncation predicts per-fire accuracy. Fourth, independent validation on other sensors and wildfire datasets, since all conclusions reported here rest on a single benchmark. Furthermore, we also aim to investigate the technique of federated learning [37,38,39] in the context of fire detection, localisation, and prediction.

## Figures and Tables

**Table 1:** Correspondence between the FirePred band index, the 27-channel sequence of the dataset publication [5], and the variable represented. Bands 1–8 of the 27-channel sequence are the VIIRS reflectance and thermal channels supplied in the separate VIIRS_Day raster.

FirePred Band	Channel	Variable
1	9	NDVI
2	10	EVI
3	11	Total precipitation
4	12	Wind speed
5	13	Wind direction
6	14	Minimum temperature
7	15	Maximum temperature
8	16	Energy release component
9	17	Specific humidity
10	18	Slope
11	19	Aspect
12	20	Elevation
13	21	Palmer drought severity index
14	22	Land cover
15	23	Total precipitation, surface
16	24	Forecast wind speed
17	25	Forecast wind direction
18	26	Forecast temperature
19	27	Forecast specific humidity

**Table 2:** Label audit across all tasks and splits. *No Dir.*: no VIIRS_Day directory present. *All NaN*: label band contains no finite pixel on any day. *<50% Days*: label band finite on fewer than half the available days. The adopted fire-level criterion excludes the first two categories only; the third is reported for the sensitivity analysis of Section 4 and those fires are retained, with unlabelled windows rejected individually.

Task	Split	Total	No Dir.	All NaN	<50% Days	Excl.	Usable
AF	train	137	13	4	3	17	120
AF	val	14	1	1	0	2	12
AF	test	17	0	2	1	2	15
BA	train	137	13	18	17	31	106
BA	val	14	1	5	0	6	8
BA	test (AF)	17	0	7	1	7	10
BA	test (US_2021)	24	0	2	4	2	22
FP	train	137	—	—	—	48	89
FP	val	14	—	—	—	6	8
FP	test	24	—	—	—	6	18

**Table 3:** Differences between the TS-SatFire reference evaluation and the protocol used in this work, grouped by whether the reference convention is stated in the dataset publication [5] or is recoverable only from the released code.

Aspect	Reference Implementation	This Work
*Stated in the dataset publication; we depart from these by choice*		
AF label rule	nan_to_num(band 7) >0	band 7 ≥7
FP target	accumulated active fire, or its union with burned area, selected per fire	clipped increment between consecutive burned-area masks
Missing values	replaced with zeros	NaN retained as unlabelled
Test stride	stride = TS; all *T* frames scored	stride 1, final frame scored
*Recoverable only from the released code*		
Aggregation	per-frame F1, averaged over frames, then fires	counts accumulated over all pixels, metric computed once
Empty ground truth	scored 1.0 (zero_division=1.0)	contributes no counts
Spatial crop	30% offset	centred
Threshold	fixed at 0.5	selected on validation

**Table 4:** Our AF model evaluated under progressively more of the reference protocol, on the reference implementation’s 30% offset crop throughout; model weights fixed. Because that crop differs from the centred one used elsewhere in this paper, no row corresponds to the configuration of Table 5.

Configuration	Test Fires	F1
Our label rule and final-frame scoring	15	0.7917
+ all frames scored	15	0.7714
+ reference label rule (band 7 >0)	15	0.7567
+ the two unannotated test fires	17	0.6912

**Table 5:** Active fire detection results. Baseline figures are reproduced from [5] under the reference evaluation protocol; our figures are measured under the protocol of Section 6.2 on the 15 annotated test fires. The two groups use different scoring conventions and are listed together for reference, not as a single ranking.

Model	TS	F1	IoU
*Published baselines, reference protocol, 17 test fires*			
U-Net (2D)	1	0.731	0.605
Att-U-Net (2D)	1	0.763	0.648
UNETR-2D	1	0.733	0.621
SwinUNETR-2D	1	0.774	0.660
GRU-3	6	0.713	0.601
LSTM-3	6	0.765	0.654
T4Fire	6	0.802	0.700
U-Net-3D	6	0.748	0.628
Att-U-Net-3D	6	0.770	0.654
UNETR-3D	6	0.811	0.706
SwinUNETR-3D	6	0.797	0.688
SwinUNETR-3D	2	0.823	0.727
*This work, protocol of Section 6.2, 15 annotated test fires*			
SpaSE-UNet3D	1	0.8520±0.0008	0.7422±0.0012
SpaSE-UNet3D	2	0.8549±0.0005	0.7466±0.0008

**Table 6:** Fire progression prediction results. Baseline figures are reproduced from [5] under the reference protocol; our figures are measured under the protocol of Section 6.2 over five independent runs. The two groups use different scoring conventions and are listed together for reference.

Model	TS	F1	IoU
*Published baselines, reference protocol*			
U-Net-3D	6	0.375	0.338
SwinUNETR-3D	6	0.374	0.331
UNETR-3D	6	0.371	0.336
Att-U-Net-3D	6	0.354	0.312
SwinUNETR-3D	2	0.366	0.321
*This work, protocol of Section 6.2*			
SpaSE-UNet3D, all 24 test fires	2	0.3845±0.0221	0.2380±0.0169
*18 fires with a usable window*	2	0.3846±0.0220	0.2381±0.0169
*16 fires with measurable progression signal*	2	0.4179±0.0124	0.2641±0.0099

**Table 7:** Training resource footprint on a single NVIDIA T4 GPU.

Task	Params	Epochs	Time (h)	Peak VRAM
AF (TS = 1)	32.88 M	100	4.2	13.2 GB
AF (TS = 2)	32.88 M	80	6.5	14.1 GB
FP (TS = 2)	24.28 M	60	3.5	13.7 GB

**Table 8:** Component ablation, AF task, TS = 2, validation split, 40 epochs per arm.

Configuration	Val F1	Δ	Δ/σ	Params
Full model (3 runs)	0.8214±0.0009	—	—	32.88 M
Full (3,3,3) convolutions	0.8205	−0.0010	1.1	89.36 M
Without SE attention	0.8219	+0.0005	0.6	32.44 M
Without deep supervision	0.8229	+0.0015	1.6	32.88 M

## Data Availability

Code, trained model checkpoints, and per-fire audit results are publicly available at https://github.com/mavroul1s/DeepFire-Forecaster (accessed on 9 August 2026).
